# The Role of Virtual Environment for Radiotherapy Training (VERT) in Medical Dosimetry Education

**DOI:** 10.1007/s13187-019-01622-2

**Published:** 2019-11-04

**Authors:** Eva Yi Wah Cheung, Maria Yuen Yee Law, Felix Cheung

**Affiliations:** 1 School of Medical Health Science, Tung Wah College, 13/F, 31 Wylie Road, Ho Man Tin, Kowloon, Hong Kong; 2 School of Public Health, Li Ka Shing Faculty of Medicine, University of Hong Kong, Patrick Manson Building, 21 Sasson Road, Pok Fu Lam, Hong Kong

**Keywords:** Dosimetry, Treatment planning, Radiation therapy, VERT, Virtual reality, Medical education, IMRT, VMAT, 3D conformal therapy

## Abstract

Medical dosimetry is an important component in training of radiation therapist, yet it is not easy for student to understand the principle of treatment planning and to be familiar with the relationship of the clinical target volume (CTV), planned target volume (PTV), and the nearby organs at risk (OARs) by just imagination. This study is conducted to evaluate whether using VERT in teaching medical dosimetry can help student to improve their learning experience. Students of cohort 2015 and 2016 were taught under TPS mode and TPS + VERT mode respectively. Direct comparison was conducted through self-evaluation survey, between two groups of students, in terms of their understanding of the concept of medical dosimetry and their level of confidence in completing different types of plans after the course. Both groups of students were able to understand the concept of medical dosimetry and able to complete 3D conformal plans after taking the course. Though, the students received TPS mode reported that they had lower level of confidence in completing the planning and required longer time for self-study and practice compared to the students who received the TPS + VERT mode. This study demonstrated that including VERT into medical dosimetry education can improve students’ learning experience, by improving their self-confidence, as well as reducing time required for their self-study and practice.

## Background

Medical dosimetry is an essential component in the radiotherapy training. Three-dimensional (3D) radiotherapy planning is a basic skill that radiation therapy students must acquire before they are qualified for professional registration. Based on different imaging modalities, including computered tomography (CT), magnetic resonance imaging (MRI), and positron emission tomography/computered tomography (PET/CT), together with the tumor and its potential spread that oncologists contoured, radiotherapy students are required to contour the organ at risks accurately. Hence, an appropriate treatment plan is developed necessary for effective treatment, and thus, a crucial part of many radiotherapy curriculum.

In Tung Wah College, MED3018 radiotherapy planning and dosimetry were designed for year 3 students of the Bachelor of Science in Radiation Therapy. Basic knowledge in oncology and radiotherapy including treatment technique, beam arrangement, and treatment side effects for cancer in different regions/systems have been delivered through the MED2110 principles of oncology and radiotherapy, MED2115 radiation oncology and radiotherapy technique 1 and MED3015 radiation oncology and radiotherapy technique 2.

The content of MED3018 is composed of 14 weeks of lectures and practical sessions. Dosimetry theory (three-dimension conformal therapy, intensity-modulated radiotherapy therapy, volumetric-modulated arc therapy), radiation-dose calculation, treatment planning system usage and quality assurance, calculation algorithm, and plan evaluation are delivered in lectures. While for practical sessions, Varian Eclipse^TM^ ver. 15.1 treatment planning system installed at Tung Wah College is used for plan generation and evaluation. This is commonly used in the oncology centers under the Hospital Authority, Hong Kong. Practicing treatment planning using Eclipse ^TM^ system allows students to be familiar with the system and the standard workflow, and enable students to perform treatment planning after they graduate from the college eventually.

Using the Eclipse^TM^ treatment planning system, the images displayed are in two-dimensional format, with the scrolling function to allow moving the CT images in volume base. This is a common display format among the imaging viewing systems, in CT or in MRI. However, most of the students find it difficult to visualize and understand the spatial relationship of organs, as well as tumors and organ at risks. As a result, they encountered difficulties in localising and contouring the tumour volume. Also, they spent long hours to practice placing the beams correctly based on the anatomical structures. In addition, plan evaluation was challenging for students, mainly due to their unfamiliarity with the anatomical spatial relationship.

In 2014, the Virtual Environment for Radiotherapy Training (VERT) was first deployed in Tung Wah College for radiotherapy treatment delivery education. Within the system, virtual treatment room was built in the software for students to practice setting up the patient for radiotherapy. These tools allowed students to acquire hands-on skills in set up and decision making as well as integrate what they have learnt in classroom.

Previous studies [1–3] showed that virtual environment helped in anatomy teaching in medical students training and in radiotherapy treatment delivery. The purpose of the project is to investigate whether virtual environment can improve the learning outcomes in medical dosimetry. The objective is to measure student’s learning outcomes based on survey results, on the basis of their perceptions about learning in different aspects. This includes speed to acquire the skills, degree of understanding in the topic, time spent to practice on treatment planning and do the same plan (exam), and confidence in treatment planning after the training. The expected research outcome is that the VR learning mode may speed up the learning, shorten the time required for them to acquire the radiotherapy planning skills, and improve their understanding in radiotherapy treatment planning.

## Method

### Ethical issues

Ethics approval for this study was granted by the research ethics sub-committee (RESC) from Tung Wah College (Ref: REC20180818). Informed consent was obtained from all individual participants included in this study. All data collected were anonymous.

### Study Design

The research was carried out through cohort study. Two cohorts (2015 and 2016 cohort) of students who enrolled the MED3018 radiotherapy planning and dosimetry course were recruited. The 2015 cohort was the control group and 2016 cohort was the measured group. All students who enrolled the course in the respective semesters were recruited such that the entire population was included.

### Data Collection

Participants were required to answer pre-course and post-course questionnaire through online Microsoft forms hosted on the Tung Wah college server. The link of the questionnaire was emailed to their college email account 2 weeks prior to the start of the course, and 2 days after the course was finished respectively. Participants gave consent prior to each questionnaire, and data was recorded anonymously.

Improvement of understanding or confidence for each participant was measured based on his/her response from pre- and post-course questionnaire. At the end of the pre-course questionnaire, participants were asked to fill in a passcode chosen by themselves. The participant had to fill in the same passcode in the post-course questionnaire, to link their data to the pre-course questionnaires.

This is an observational study, no intervention was employed to change the teaching within the same cohort.

### Teaching Mode

The same course content was delivered to both the control group and the measured group, in treatment planning system learning mode (TPS mode) and treatment planning system + VERT learning mode (TPS + VERT mode) respectively. The lectures were highly similar and they were taught by the same person. The students did not received prior lecture in radiotherapy treatment planning. All students attended 6-week clinical training in planning prior to the course started.

The TPS mode used Varian Eclipse ^TM^ version 15.1 treatment planning system for contouring organ at risks in the chest and pelvis regions, 3D conformal field-in-field plan generation and evaluation for ca breast and ca rectum cases, and IMRT and VMAT plan demonstration for ca prostate cases.

The content of TPS + VERT mode was the same as the TPS mode, with an allocation of 15 min within each practical session, for demonstration in the VERT system of contouring organ at risks in chest and pelvis regions, 3D conformal field-in-field plan generation, plan evaluation for ca breast and ca rectum cases, and IMRT and VMAT plan demonstration for ca prostate cases.

### Questionnaire Design

The questionnaire composed of four parts: (1) demographics and prior experience to medical dosimetry, (2) knowledge in planning, (3) satisfaction to the TPS, and the (4) learning experience.

### Statistical Analysis

For part 1 questions related to demographics, Student’s *t* test was used. For part 2–4 questions, Likert scores 1–5 were assigned and coded using Microsoft Excel, and analysed using IBM SPSS version 25.0 (Table 1).

**Table 1:** Likert scale used in the questionnaire [4]

1	2	3	4	5
Very limited	Limited	Neutral	Good	Very Good
Strongly disagree	Disagree	Neutral	Agree	Strongly agree

Based on the pre- and post-course questionnaire, the improvement in understanding and confidence of each student could be assessed. Chi square test (Fisher’s exact test-2 sided) was used to determine the statistical significance.

## Result

Of a total of 10 students enrolled in the TPS group, 9 students took part in both questionnaires. For the TPS + VERT group, all 16 students took part in both questionnaires.

### Prior Experience in Medical Dosimetry

The majority of participants in both groups reported having planning experience prior to the course (78% and 81% respectively) during the previous clinical placement. In the TPS group, 44% received more than 50-h planning experience. While 77% of TPS + VERT group received less than 10 h training in planning.

### Student’s Knowledge About Medical Dosimetry (outcome 1)

To determine whether the student improved their understanding and knowledge in RT planning concept within their corresponding teaching module, mean score of their self-reported confidence level and percentage of student who have improved confidence were calculated and compared.

In the pre-course questionnaire, the TPS + VERT group student reported less confidence in all aspects when compared to TPS group (mean score from 1.44 ± 0.629 to 3.13 ± 1.025 vs 2.33 ± 0.707 to 3.66 ± 0.707).

In the post-course questionnaire, the confidence level of both groups attained similar results, with mean score from 3.56 ± 0.882 to 4.33 ± 0.707 vs 3.69 ± 0.793 to 4.38 ± 0.5 in TPS and TPS + VERT group respectively. The mean score difference in TPS + VERT group was much higher than that in TPS group in all aspects, from 0.87 to 2.51 vs 0.45 to 1.56 respectively.

After the course, all students reported improved confidence in all aspects in RT planning. In particular, over 80% of student improved their confidence in understanding in 3D conformal radiotherapy (88.9% and 100% for TPS and TPS + VERT group respectively), competence in using DVH to evaluate TV and organ at risks (both are 88.8% and 81.3 for TPS and TPS + VERT group respectively). While for more complex techniques (IMRT and VMAT), the improvement is less when compared to other aspects. Only 55% and 62.5% of student reported improved confidence in IMRT and 66.6% and 62.5% in TPS and TPS + VERT group respectively.

However, all the above results were statistically not significant based on two-tailed tests with a 5% type-I error rate (Table 2).

**Table 2:** Likert scale of the pre- and post-course questions—the student’s knowledge about medical dosimetry [4]

	Likert scale							
	TPS group (mean score)				TPS + VERT group (mean score)			
Core questions	Pre-course	Post-couse	Mean score difference	Agree + strongly agree	Pre-course	Post-couse	Mean score difference	Agree + strongly agree
Q1 How would you rate your current knowledge towards radiotherapy treatment planning?	3.33 ± 0.866	3.78 ± 0.833	0.45	77.8%	1.69 ± 0.704	3.81 ± 0.834	2.12	68.8%
Q2 How would you rate your current understanding in 3D conformal radiotherapy?	3.11 ± 0.928	4.11 ± 0.928	1.0	88.9%	1.87 ± 0.806	4.38 ± 0.5	2.51	100%
Q3 How would you rate your current understanding in intensity modulated radiotherapy (IMRT)?	2.33 ± 0.707	3.56 ± 0.882	1.23	55.5%	1.50 ± 0.516	3.69 ± 0.793	2.19	62.5%
Q4 How would you rate your current understanding in volumetric modulated arc therapy (VMAT)?	2.33 ± 0.86	3.89 ± 0.782	1.56	66.6%	1.44 ± 0.629	3.75 ± 0.856	2.31	62.6%
Q5 How would you rate your competence in using dose volumetric histogram (DVH) to evaluate the target volume coverage?	3.66 ± 0.707	4.33 ± 0.707	0.67	88.8%	2.12 ± 1.025	3.94 ± 0.574	1.82	81.3%
Q6 How would you rate your competence in using dose volumetric histogram (DVH) to evaluate the dose to organ at risks?	3.44 ± 0.882	4.11 ± 0.928	0.67	88.8%	2.31 ± 0.946	3.94 ± 0.574	1.63	81.3%
Q7 How would you rate your competence in evaluating the radiotherapy plans based on the 2D display of CT images, based on the contoured target volume and organ at risks?	3.11 ± 1.054	4.0 ± 0.707	0.33	77.8%	3.13 ± 1.025	4.0 ± 0.632	0.87	81.3%
Q8 How useful will the CT images (2D display) to your understanding of anatomy?	3.55 ± 0.882	4.0 ± 0.707	0.45	77.8%	2.56 ± 0.892	3.87 ± 0.719	1.31	81.3%
Q9 Rate your current knowledge on how anatomy connects to radiotherapy planning theory?	3.44 ± 0.726	4.0 ± 0.866	0.56	88.8%	2.63 ± 1.088	4.0 ± 0.816	1.37	81.3%

### Students’ Perspective Towards the Treatment Planning System (outcome 2)

All students reported improved confidence in controlling the treatment planning system and they like to use the planning system (88.9% in TPS group and 81.3% in TPS + VERT group). For TPS group, 88.9% of students found that the planning system is easy to use, while only 62.6% for TPS + VERT group. However, the results were not statistically significant (Table 3).

**Table 3:** Likert scale of the pre- and pos-course questions—students’ perspective towards the treatment planning system [4]

	Likert Scale							
	TPS Group (mean score)				TPS + VERT Group (mean score)			
Core questions	Pre-course	Post-couse	Mean score difference	Agree + strongly agree	Pre-course	Post-couse	Mean score difference	Agree + strongly agree
Q1 I expect I can control the equipment as I needed when using the planning system	3.89 ± 0.601	4.11 ± 0.601	0.22	88.9%	3.50 ± 0.816	4.06 ± 0.68	0.56	81.3%
Q2 I expect I like using the planning system	3.88 ± 0.601	3.89 ± 0.782	0.01	88.9%	3.62 ± 0.806	4.0 ± 0.632	0.38	81.3%
Q3 The planning system may be easy to use	3.44 ± 1.014	3.88 ± 0.782	0.44	88.9%	3.06 ± 0.998	3.63 ± 1.025	0.57	62.6%
Q4 Technical issues will make using the planning system difficult	3.77 ± 0.833	3.56 ± 0.726	− 0.021	44.4%	3.56 ± 0.892	3.19 ± 0.834	− 0.37	43.8%

### Students’ Perspective in Their Learning Module (outcome 3)

Overall, both groups reported improvements in all aspects within the respective learning mode, while higher percentage were found in TPS + VERT group when compared to TPS group. In particular, TPS + VERT group had over 90% students reported statistically significant higher score in improvement of learning anatomy and better control in planning system and found easier in learning RT planning (Table 4).

**Table 4:** Likert scale of the students’ perspective in their learning module [4]

	TPS group		TPS + VERT group	
Core questions	Mean score	Agree + strongly agree	Mean score	Agree + strongly agree
Q1 The learning mode enabled me to think more about radiotherapy planning procedure	3.88 ± 0.601*	77.8%	4.44 ± 0.892*	87.5%
Q2 The learning mode had a positive effect on my ability in radiotherapy planning	3.88 ± 0.601*	77.8%	4.69 ± 0.602*	93.8%
Q3 The learning mode helped me to learn as I can repeat the activities until I was happy with the results	3.88 ± 0.601*	77.8%	4.56 ± 0.629*	93.8%
Q4 The learning mode helped me to learn faster in radiotherapy planning	3.77 ± 0.441*	77.8%	4.5 ± 0.632*	93.8%
Q5 The learning mode was appropriate in teaching radiotherapy planning, I learnt most of the techniques that required in clinical situation.	3.66 ± 0.707*	77.8%	4.5 ± 0.632*	93.8%
Q6 The learning mode made the learning of radiotherapy planning easier	3.66 ± 0.707*	77.8%	4.56 ± 0.629*	93.8%
Q7 The learning mode helped me in learning anatomy	3.66 ± 0.866*	66.7%	4.69 ± 0.479*	100%
Q8 The learning mode helped me in better control in planning system	3.66 ± 0.5*	66.7%	4.50 ± 0.632*	93.8%
Q9 The learning mode helped me in understanding dose volume histogram (DVH)	3.56 ± 0.882*	55.5%	4.06 ± 0.68*	81.3%
Q10 The learning mode helped me in understanding plan evaluation	4.00 ± 0.707	77.8%	4.56 ± 0.512	100%
Q11 Overall, I would like to use the learning mode in radiation dosimetry course	4.11 ± 0.601	88.9%	4.63 ± 0.619	93.8%

*p < 0.05 (two-sided Fisher’s exact test for significance

## Discussion

In health care professional education, simulation is one of essential tools for students to acquire skills, knowledge, and behaviors associated with patient care. A recent systematic review identified 609 studies, which related to technology-enhanced simulation for healthcare professional education. It concluded that positive results have attained in skills outcome, time to acquire skills and knowledge, learner’s performance, and effects on patient care [5].

VERT system has been applied globally since 2007 for RT training [6]. Wide varieties of studies have been done to investigate the value of VERT in training RT students for treatment delivery [7, 8] and simulation [9, 10]. For VERT application in medical dosimetry, VERT has been used to demonstrate dosimetric effects in different treatment techniques, so as to evaluate the possible patient side effects [11]. However, contents of the sessions were limited in the report.

In Leong et al study, cross-over study design was used to investigate student and teacher’s perspectives [12]. As all students were exposed to both teaching modules, the conceptual understanding of RT may be affected by previous learning modules; the learning experience may be influenced, and consecutively their questionnaire responses. Here, cohort study design was employed, mainly to fit for the small sample size. The course content for TPS group and the TPS + VERT group is the same within the cohort. This was to ensure both classes experienced only one teaching module and their responses could be compared directly. Also, their assessment result would not be influenced so as to maintain the fairness.

A short online questionnaire was chosen to minimize the commitment for the participants [13]. All questionnaires were kept at the college server for security. Except for questions related to demographics, questions were designed based on five-point Likert scale. Target participants (college students) were familiar with the Likert scale and hence, improved the response rate [14]. Free text questions were not included in this study to improve the result consistency, while qualitative feedback was not able to be collected.

Both TPS and TPS + VERT groups improved their knowledge after the course in all aspects. The confidence level of the TPS + VERT group was much lower than the TPS group in all aspects prior to the course started. This may be due to their limited experience of medical dosimetry during previous clinical placement (81% of students had planning experience, with 77% less than 10-h planning experience). After the course, both groups attained similar level of confidence and knowledge. All results were statistically not significant, indicating that the improvement of knowledge was independent of the mode of learning.

After the course, both TPS and TPS + VERT groups improved their confidence in using the treatment planning system and like using it. Again, the results in this part were statistically not significant; the improvement was independent to the mode of learning.

Both TPS and TPS + VERT group reacted positively in all aspects within their mode of learning, indicating that the course helped them to learn RT planning, no matter in which learning mode. The mean score in TPS + VERT group is higher which demonstrated their high satisfaction in the learning mode. All students in this group agreed that TPS + VERT module helped them in learning anatomy, having better control in planning system, finding easier and faster learning in RT planning. In view of the fact that the course content of both learning mode were the same, the only difference is the demonstration and explanation of plan using VERT system or not. Using the VERT is a cost effective tool that helped student learning in RT planning and improved their learning experience without changing the radiotherapy curriculum.

### Study Limitation

The sample size of this study was small. Both the trends on survey response (mean score in Likert scale) and the statistically based evidence were investigated. While nearly all students were recruited (90% in control and 100% in measured group), the effect of sampling was minimized.

Our baseline assumption was that both TPS and TPS + VERT group had the same level of RT background before the course, and thus, the learning of RT planning was only contributed by the course.

Improvement in satisfaction was from TPS and TPS + VERT group. Learning effects from other platforms, e.g., YouTube were not included.

## Conclusion and Future Direction

Both groups demonstrated an improvement in RT medical dosimetry after taking the course in their respective learning mode and were satisfied in using treatment planning system. However, TPS + VERT group commented that VERT helped them in learning RT planning, in particular, anatomy, control in planning system, understanding DVH, and treatment technique. In general, they found that it was easier for them to acquire the skills, faster in learning. The overall experience in TPS + VERT group was better when compared to the TPS group, suggesting that the VERT should be incorporated in the medical dosimetry training in radiotherapy.
