# Correction to: The Role of Virtual Environment for Radiotherapy Training (VERT) in Medical Dosimetry Education

**DOI:** 10.1007/s13187-023-02306-8

**Published:** 2023-05-04

**Authors:** Eva Yi Wah Cheung, Maria Yuen Yee Law, Felix Cheung

**Affiliations:** 1 School of Medical Health Science, Tung Wah College, 13/F, 31 Wylie Road, HoMan Tin, Kowloon, Hong Kong; 2 School of Public Health, Li Ka Shing, Faculty of Medicine, University of Hong Kong, Patrick Manson Building, 21 Sasson Road, Pok Fu Lam, Hong Kong

**Correction to: Journal of Cancer Education**

10.1007/s13187-019-01622-2

The article The Role of Virtual Environment for Radiotherapy Training (VERT) in Medical Dosimetry Education, written by Eva Yi Wah Cheung, Maria Yuen Yee Law & Felix Cheung was originally published electronically on the publisher’s internet portal on 4 November 2019 without open access. With the author(s)’ decision to opt for Open Choice the copyright of the article changed on April 2023 to © The Author(s) 2019 and the article is forthwith distributed under a Creative Commons Attribution 4.0 International License, which permits use, sharing, adaptation, distribution and reproduction in any medium or format, as long as you give appropriate credit to the original author(s) and the source, provide a link to the Creative Commons license, and indicate if changes were made. The images or other third party material in this article are included in the article’s Creative Commons licensee, unless indicated otherwise in a credit line to the material. If material is not included in the article’s Creative Commons license and your intended use is not permitted by statutory regulation or exceeds the permitted use, you will need to obtain permission directly from the copyright holder. To view a copy of this license, visit http://creativecommons.org/licenses/by/4.0.

The original article has been corrected.

**Open Access** This article is licensed under a Creative Commons Attribution 4.0 International License, which permits use, sharing, adaptation, distribution and reproduction in any medium or format, as long as you give appropriate credit to the original author(s) and the source, provide a link to the Creative Commons licence, and indicate if changes were made. The images or other third party material in this article are included in the article's Creative Commons licence, unless indicated otherwise in a credit line to the material. If material is not included in the article's Creative Commons licence and your intended use is not permitted by statutory regulation or exceeds the permitted use, you will need to obtain permission directly from the copyright holder. To view a copy of this licence, visit http://creativecommons.org/licenses/by/4.0/.

